# Unraveling skeletal muscle senescence in an *in vitro* cell culture model

**DOI:** 10.3389/fragi.2026.1866906

**Published:** 2026-09-18

**Authors:** Kayo Moreira Bagri, Morgana Teixeira Lima Castelo-Branco, Claudia Mermelstein

**Affiliations:** 1 Instituto de Ciências Biomédicas, Universidade Federal do Rio de Janeiro, Rio de Janeiro, Brazil; 2 Hospital Universitário Clementino Fraga Filho, Universidade Federal do Rio de Janeiro, Rio de Janeiro, Brazil

**Keywords:** cytosine arabinoside, FGF, lysosomes, muscle, ROS, senescence, p53, desmin

## Abstract

**Introduction:**

Skeletal muscles are crucial for voluntary movement, posture, breathing, joint stability, and therefore are essential for maintaining overall health and quality of life. Aging, sarcopenia, cancer, immobilization, and several muscle pathologies can have an impact on muscle function, and muscle cell senescence is associated with these conditions.

**Methods:**

Here we aimed to develop a primary cell culture model to study skeletal muscle cell senescence. We induced cell cycle arrest in cultured embryonic chick muscle cells using a low concentration of cytosine arabinoside (Ara-C) for 9 days.

**Results:**

Our results show that Ara-C was able to induce several cellular senescence-associated bone fide characteristics, including reduced cell proliferation, increased β-galactosidase expression, increased reactive oxygen species production (ROS), increased p53 and p21 expression, increased muscle fibroblast cell size and nuclei numbers, increased lysosomal activity, decreased muscle differentiation (decreased muscle fiber size and decreased desmin expression), and increased secretion of IL-6, TNFα and TGFβ. Interestingly, inhibition of fibroblast growth factor receptor recovers the AraC-induced senescent phenotype in myogenic cells.

**Conclusion:**

This new experimental model could be used to deepen our understanding of the molecules and signaling pathways associated with the initiation and establishment of skeletal muscle senescence. Furthermore, this model could be used to test new drugs and therapeutic interventions aiming the amelioration of senescence-associated conditions.

## Introduction

Senescence is a cellular process in which cells cease to divide and acquire several characteristics, including metabolic adaptations, morphological changes, intense secretory profile, changes in cell surface markers, chromatin reorganization, and increased lysosomal compartments (Huang et al., 2022). Cellular senescence occurs during normal physiological conditions, including embryogenesis, but can be associated with pathological conditions (de Magalhães, 2024). How cells become senescent, how senescence is maintained, and how different is the senescence program among diverse cell types are not fully comprehended (Ogrodnik et al., 2024). Skeletal muscle cells are a particularly interesting cell type where cellular senescence has an intimate connection with aging and sarcopenia (He et al., 2022). Sarcopenia is a muscle pathology characterized by the progressive loss of muscle mass and strength (Sayer et al., 2024). Here, we developed a new *in vitro* model to deepen our understanding of skeletal muscle cell senescence. We induced cell cycle arrest in muscle cells using a low concentration (150 nM) of cytosine arabinoside (Ara-C) for 9 days. Primary skeletal muscle cell cultures were obtained from 11-day-old chick embryos. The choice for the *in vitro* chick muscle cell culture is related to the high level of cell differentiation of the autonomous chick myogenic program, which is independent of serum withdrawal and strengthens the power of the model (Choi et al., 1990). Chick myotubes can be formed with hundreds of nuclei, whereas myotubes from commonly used muscle cell lines (e.g., C2C12) usually contain around a dozen nuclei (Costa et al., 2021). Another important feature of chick myogenic cultures is that they comprise myoblasts, myotubes and fibroblasts. Fibroblasts are important to muscle cell differentiation in culture, since they secrete many components of the extracellular matrix, such as collagen, laminin, and fibronectin, as well as numerous growth factors and cytokines (Chapman et al., 2016). Their presence in chick muscle cell cultures mimic the *in vivo* muscle tissue environment. Our results show that Ara-C induced several cellular senescence-associated bone fide characteristics, including reduced cell proliferation, increased beta-galactosidase expression, increased reactive oxygen species (ROS) production, increased p53 and p21 expression, increased muscle fibroblast cell size, increased lysosomal activity, decreased muscle differentiation (decreased myotube size), and increased secretion of IL-6, TNFα and TGFβ. The collection of these results shows that low concentrations of Ara-C induce a skeletal muscle cell senescence phenotype. This new experimental model on muscle cell senescence could be a valuable tool for future studies aiming to understand the molecular and cellular basis governing several muscle degeneration conditions, such as muscle disuse, aging and sarcopenia.

## Methods

### Antibodies and probes

The following antibodies were used in this study: rabbit polyclonal anti-desmin (code D8281, Sigma-Aldrich, United States of America), rabbit polyclonal anti-p53 (code SAB4503001, Sigma), rabbit monoclonal anti-p21 (code 2,947, Cell Signaling), mouse monoclonal anti-α-tubulin (code T-9026, Sigma), mouse monoclonal anti-beta-actin (clone A5316, Sigma), mouse monoclonal anti-flotillin-2 antibody (clone 29, BD Transduction Labs, United States of America), and rabbit polyclonal anti-phospho-histone H3 (code 06-570, Millipore, United States of America). DNA-binding probe DAPI (4,6-Diamino-2-phenylindole), 546-Phalloidin, LysoTracker Yellow HCK-123 (code L12491), H2DCFDA (2,7-Dichlorodihydrofluorescein diacetate), Alexa Fluor 488-goat anti-rabbit IgG antibodies, and Alexa Fluor 546-goat anti-mouse IgG antibodies were from Invitrogen (Thermo Fisher Scientific, United States of America). Hoechst 33342 (bisBenzimide H 33342 trihydrochloride, code 14533) was from Sigma.

### Primary cultures of chick myogenic cells

This study using chick embryos was approved by the Ethics Committee for Animal Care and Use in Scientific Research from the Federal University of Rio de Janeiro and received the approval number: 107/25. Primary myogenic cells were prepared as described previously (Mermelstein et al., 2005). Briefly, pectoral muscle tissue was removed from six 11-day old chicken embryos (Granja Tolomei, Brazil). After separation of the connective tissue under a dissecting microscope, muscle tissue was minced with surgery knives and digested with trypsin (0.005%) at 37 °C in a 5% CO_2_ incubator for 25 min. To stop digestion, a small volume of 8-1-5 medium (Minimum Essential Medium with 10% horse serum, 5% chick embryo extract, 1% L-glutamine, and 1% penicillin-streptomycin, all from Gibco, Brazil, except the chick embryo extract that was made in our lab) was added to the sample and then centrifuged at approximately 300 × *g* for 5 min. After discarding the supernatant, the pellet was suspended in 8-1-5 medium and filtered to produce a mononucleated cell suspension. Isolated mononucleated cells were plated onto collagen or 0.1% gelatin coated Aclar coverslips (Pro-Plastics, United States of America) at an initial density of 5.5 x 10^5^ cells/35 mm culture dishes in 2 mL of 8-1-5 medium and grown in a humidified 5% CO_2_ atmosphere at 37 °C.

The percentage of myoblasts and fibroblasts in these chick myogenic cell cultures was calculated by the triple-labeling of 24-h cultures with DAPI (nuclear staining), anti-desmin antibody (a muscle-specific marker) and anti-flotillin-2 antibody (a marker of chick muscle fibroblast cells, Possidonio et al., 2014), and subsequently counting the number of desmin-positive mononucleated cells and the number of flotillin-2-positive cells out of the total number of cells in the field. On average, myoblasts made up 80% of each culture and fibroblasts comprised 20%. Immunolabeling cells with antibodies against flotillin-2 confirmed that all non-myogenic cells in these cultures are fibroblasts.

Some chick myogenic cells were treated with 5 or 10 μM of SU-5402 (Sigma-Aldrich), the Fibroblast Growth Factor receptor (FGFr) inhibitor 3-[4-Methyl-2-(2-oxo-1,2-dihydro-indol-3-ylidenemethyl)-1H-pyrrol-3-yl]-propionic acid.

### Senescence induction and senescence markers

To induce cellular senescence, chick myogenic cell cultures were plated at an initial density of 5.5 x 10^5^ cells/35 mm culture dishes and treated with 150 nM arabinocytosine (Ara-C) for 3 days during primary culture (P0). Then, cells were trypsinized and plated at 0.5 x 10^5^ cells/35 mm dishes for 6 days (P1). Then, cells were washed and maintained in 8-1-5 medium without Ara-C for the next 6 days (P2). Ara-C is a synthetic antimetabolite that functions as a structural analogue of cytidine. Ara-C is an S-phase specific drug that inhibits DNA synthesis and cell proliferation (Curt et al., 1987). It has been shown that low doses of Ara-C can interfere with cell differentiation (Tomic et al., 2022). We analyzed the following senescence markers in chick myogenic cells: beta-galactosidase (β-gal), p53, reactive oxygen species (H2DCFDA), intracellular acid organelles (acridine orange), lysosomes (Lysotracker), Sudan Black, phospho-histone H3 (pH3), IL-6, TNF-α, TGF-β, desmin (muscle differentiation marker), number of nuclei within cells (DAPI and Hoechst), and cell size.

### SA-β-galactosidase activity

SA-β-gal activity was detected according to the protocol described by Dimri et al. (1995). Briefly, cultured cells were washed twice with phosphate-buffered saline (PBS) and fixed for 3 min at room temperature in 2% formaldehyde and 0.2% glutaraldehyde in 0.1 M phosphate buffer. Following fixation, cells were washed 2-3 times with PBS and incubated at 37^∘^C in the absence of CO_2_​ for 12–16 h with a fresh staining solution. The staining solution consisted of 1 mg/mL 5-bromo-4-chloro-3-indolyl β-D-galactopyranoside (X-gal), 40 mM citric acid/sodium phosphate buffer (pH 6.0), 5 mM potassium ferrocyanide, 5 mM potassium ferricyanide, 150 mM NaCl, and 6 mM MgCl_2_​. Development of cytoplasmic blue and purple color was assessed as a marker of cellular senescence.

### Analysis of the production of reactive oxygen species

Production of reactive oxygen species (ROS) was analyzed with the cell-permeant 2′,7′-dichlorodihydrofluorescein diacetate (H2DCFDA), which is a chemically reduced form of fluorescein used as an indicator for ROS in cells. Briefly, control myogenic cells or senescent cells were washed three times with PBS and incubated with 600 µM H2DCFDA for 30 min at 37 °C. The redox state of live cells was monitored by detecting the increase in fluorescence (emission: 517–527 nm). Hoechst was used as a live nuclear counterstain.

### Sudan black B (SBB) staining for lipofuscin detection

Lipofuscin accumulation was assessed using Sudan Black B (SBB) staining according to the optimized protocol by Davan-Wetton and Montero-Melendez (2024). Briefly, a 0.7% SBB solution was prepared by dissolving SBB powder in 70% ethanol with overnight stirring. Immediately prior to staining, the solution was sequentially filtered through a 70 μm cell strainer, a 0.45 μm syringe filter, and a 0.22 μm syringe filter to eliminate dye aggregates. Fixed cells were rinsed with 70% ethanol and incubated with the filtered SBB solution for 1h at room temperature. To avoid non-specific dye precipitation on the cell monolayer, the staining step was performed under constant agitation on a plate shaker. Cells were subsequently washed with 50% ethanol and PBS to remove excess dye and imaged using bright-field microscopy or fluorescence microscopy in the Far-Red channel.

### Cell viability assay

Cell viability was determined using 3-(4,5-dimethyl-2-thiazyl)-2,5-diphenyl-2H-tetrazolium bromide (MTT) reagent (Sigma-Aldrich). Briefly, cells were plated at an initial density of 10^5^ cells per well in 24-well plates and incubated at 37 °C and 5% CO_2_. After 96 h some cultures were treated with Ara-C at a final concentration of 150 nM and further incubated for 72 h. After treatment, the supernatant of each well was removed, and cells were washed twice with medium. Then, 250 μL of MTT solution (5 mg mL^–1^ in MEM without phenol red) were added to each well and incubated for 3 h at 37 °C, 5% CO_2_. The resultant formazan crystals were dissolved in dimethyl sulfoxide (250 μL) and absorbance intensities were measured in a microplate reader (FlexStation Reader, Molecular Devices, United States of America) at 570 nm. All experiments were performed in triplicate, and cell viability was expressed as a percentage relative to the untreated control cells.

### Immunofluorescence and digital image acquisition

Chick myogenic cells were rinsed with PBS and fixed in 4% paraformaldehyde for 15 min. Then, they were permeabilized in PBS with 0.5% Triton X-100 (PBS/T) for 30 min, blocked in PBS with 1% bovine serum albumin and 5% fetal bovine serum (blocking solution) for 30 min, and incubated overnight at 4 °C with primary antibodies (all diluted 1:100 in blocking solution) against p53, phospho-histone H3, desmin, α-tubulin and flotillin-2. After incubation, cells were washed for 30 min in PBS and incubated for 1 h at 37 °C with Alexa Fluor-conjugated secondary antibodies (all diluted 1:100 in PBS). Nuclei were labeled with DAPI (fixed cells) or Hoechst (live cells). Cells were mounted in Prolong Gold (Molecular Probes) and examined with either an Axiovert 100 microscope (Carl Zeiss, Germany) coupled to an Olympus DP71 high-resolution camera, or a DSU Spinning Disk confocal scanner mounted on an inverted fluorescent microscope (Olympus, Japan). Images of control and senescent cells were acquired using the same parameters in the fluorescence microscopes. Control experiments with no primary antibodies showed only faint background staining (data not shown). Images were adjusted for brightness and contrast using Fiji software (Schindelin et al., 2012) and figure plates were mounted with Adobe Photoshop software (Adobe Systems Inc., United States of America).

### Quantification of chick cell cultures

The quantification of the number of myoblasts, number of fibroblasts, number of nuclei within myotubes, number of nuclei within fibroblasts, total number of nuclei, myoblast fusion index, myotube width, area of fibroblasts and area of muscle cells was performed in chick myogenic cells immunolabeled for the muscle-specific marker desmin (Costa et al., 2004) and the nuclear dye DAPI using Fiji software. DAPI and desmin double staining enables the identification of myoblasts by the presence of desmin and fibroblasts by differences in nuclear morphology and DAPI fluorescence intensity. More specifically, muscle fibroblasts show large, flattened, pale nuclei and negative cytoplasm staining for desmin, whereas myoblasts are desmin-positive cells with small, round, and bright nuclei (Costa et al., 2021). All data were quantified from fifty randomly chosen microscopic fields collected from three independent experiments.

### Immunoblotting

The protein concentration of cell culture homogenates was determined with the Bradford assay (Pierce™ Bradford Protein Assay Kit, ThermoFisher) and denatured with Laemmli buffer at 95 °C for 5 min. Samples were separated on 12.5% sodium dodecyl sulfate-polyacrylamide gel electrophoresis (SDS-PAGE) and transferred onto 0.45 μm Polyvinylidene fluoride (PVDF) membranes (code IEVH85R, Millipore, Burlington, MA, United States of America). Precision plus protein kaleidoscope prestained protein standards (code 1610375, Bio-Rad Laboratories) were used as molecular weight markers. The proteins immobilized on the membranes were immediately blocked for 1-h at room temperature with a solution of 5% nonfat dry milk in Tris buffered saline-Tween 20 (0.001%) (TBS-T). PVDF membranes were incubated overnight at 4 °C with antibodies against desmin (dilution 1:4000), beta-actin (dilution 1:1000), p53 (dilution 1:500) and p21 (dilution 1:2000). The next day, membranes were incubated with horseradish peroxidase-conjugated secondary antibodies (1:2000, Invitrogen, code 31460 and code 31430) and antibody-bound bands were visualized with the Super Signal™ West Pico PLUS Chemiluminescent Substrate using a ChemiDoc™ XRS + System with Image Lab™ Software (code 1708265, Bio-Rad, United States of America).

## ELISA

The levels of IL-6, TNF-α and TGF-β in chick myogenic cell cultured media were determined by enzyme-linked immunosorbent assay (ELISA) protocol, using commercial murine ELISA kits standardized by R&D Systems (United States of America), catalog number DY406, DY410 and DY1679, respectively. Although the overall sequence homology between avian and mammalian cytokines is generally low (25%–45%) (Kaiser et al., 2001; Wigley and Kaiser, 2003), this divergence does not preclude immunological cross-reactivity. Antibody binding relies on the conservation of specific structural epitopes rather than the entire protein sequence. This cross-reactivity of mouse ELISA cytokine kits against chicken samples has been previously validated in embryonic chick myogenic cell cultures (Possidonio et al., 2011). Based on this methodological precedent, we utilized mammalian ELISA kits to assess the relative cytokine profiles in our experimental model. Conditioned media were collected from control and senescent experimental conditions. Samples were centrifuged and the supernatants were used for cytokine concentrations. Briefly, the anti-mouse capture monoclonal antibodies (R&D Systems, United States of America) were absorbed on a polystyrene 96-well plate (Maxisorb, NUNC, Denmark), and the cytokine present in the sample was bound to the antibody-coated wells. The recombinant mouse of each cytokine (R&D Systems, United States of America) was added to standard wells. The biotinylated anti-mouse detecting mAb (R&D Systems) was added to bind the cytokine captured by the first antibody. After washing, streptavidin-horseradish peroxidase (R&D Systems) was added to the wells to detect the biotinylated detecting antibody, and finally a tetramethyl benzidine chromogenic solution (eBioscience, United States of America) was added as a substrate, and a colored product formed in proportion to the amount of each cytokine present in the samples was stopped with 1 N HCl. Absorbance was read using 450 nm as wavelength. All samples were analyzed in triplicate. To account for differences in cell density between control and senescent cultures, cytokine levels measured by ELISA were normalized to the total cell number per well. Parallel cultures were fixed with 4% paraformaldehyde, and nuclei were stained with the nuclear dye DAPI to quantify cell numbers. The absolute cytokine concentration (pg/mL) was divided by the estimated total cell count for each sample. Final data are expressed as picogram of cytokine per million cells (pg/10^6^ cells).

### Statistical analysis

Statistical analyses were performed using the GraphPad Prism 8 software (GraphPad Software Inc., United States of America). Biological and technical replicates: six 11-day old chicken embryos were used to produce five 35-mm muscle cell culture dishes per experiment. Fifty randomly chosen microscopic fields were obtained from each culture dish. Three independent experiments (N = 3) were performed for each analysis (SA-β-gal, ROS, ELISA, SBB, etc.). Data normality was assessed before applying parametric statistical tests and the results are shown in Supplementary Figure S1. False Discovery Rate test (FDR) was done to analyze p values from the quantification of chick cell cultures (Supplementary Figure S2). To assess the level of statistical significance Unpaired t-test and One-Way ANOVA followed by Tukey’s multiple comparisons test were used with p < 0.05. The results were expressed as mean values ± standard deviation (SD). *P ≤ 0.05, **P ≤ 0.01, ***P ≤ 0.001, ****P ≤ 0.0001.

## Results and discussion

Here, we aimed to develop a new *in vitro* model to study skeletal muscle cell senescence. We induced cell cycle arrest in muscle cells grown in culture by exposing them to a low concentration of cytosine arabinoside (Ara-C), a S-phase specific drug that inhibits DNA synthesis and cell proliferation (Curt et al., 1987). Primary cultures of embryonic chick myogenic cells were grown for 96 h, treated with 150 nM of cytosine arabinoside (Ara-C) for 72 h and the expression of beta-galactosidase (β-gal) was assessed. We initiated with β-gal the analysis of senescence markers, since β-gal is considered a gold standard marker for cellular senescence (Debacq-Chainiaux et al., 2009). Chick myogenic cells treated with Ara-C showed a more than 4-fold increase in the expression of β-gal (Figures 1A–F). Interestingly, β-gal staining (in purple) was observed in mononucleated cells (myoblasts and fibroblasts) and in multinucleated myotubes treated with Ara-C (Figures 1D,E), indicating that the senescence phenotype was observed in all three major cell types present in chick myogenic cell cultures. Since a low dose of Ara-C (150 nM) was able to induce senescence in these chick muscle cells, all further results will be labeled as control (untreated cells) or senescent (AraC-treated cells). We also analyzed whether 150 nM of Ara-C, besides inducing senescence, could also interfere with cell viability. Cells were treated with 150 nM Ara-C for 3 days during primary culture (P0). Then, cells were trypsinized and plated again for 6 days (P1), washed and maintained in culture medium without Ara-C for the next 6 days (P2). MTT assay showed no significant interference of the low dose of Ara-C in cell viability in P0 and in P2 cultures (Figure 2).

**FIGURE 1:**
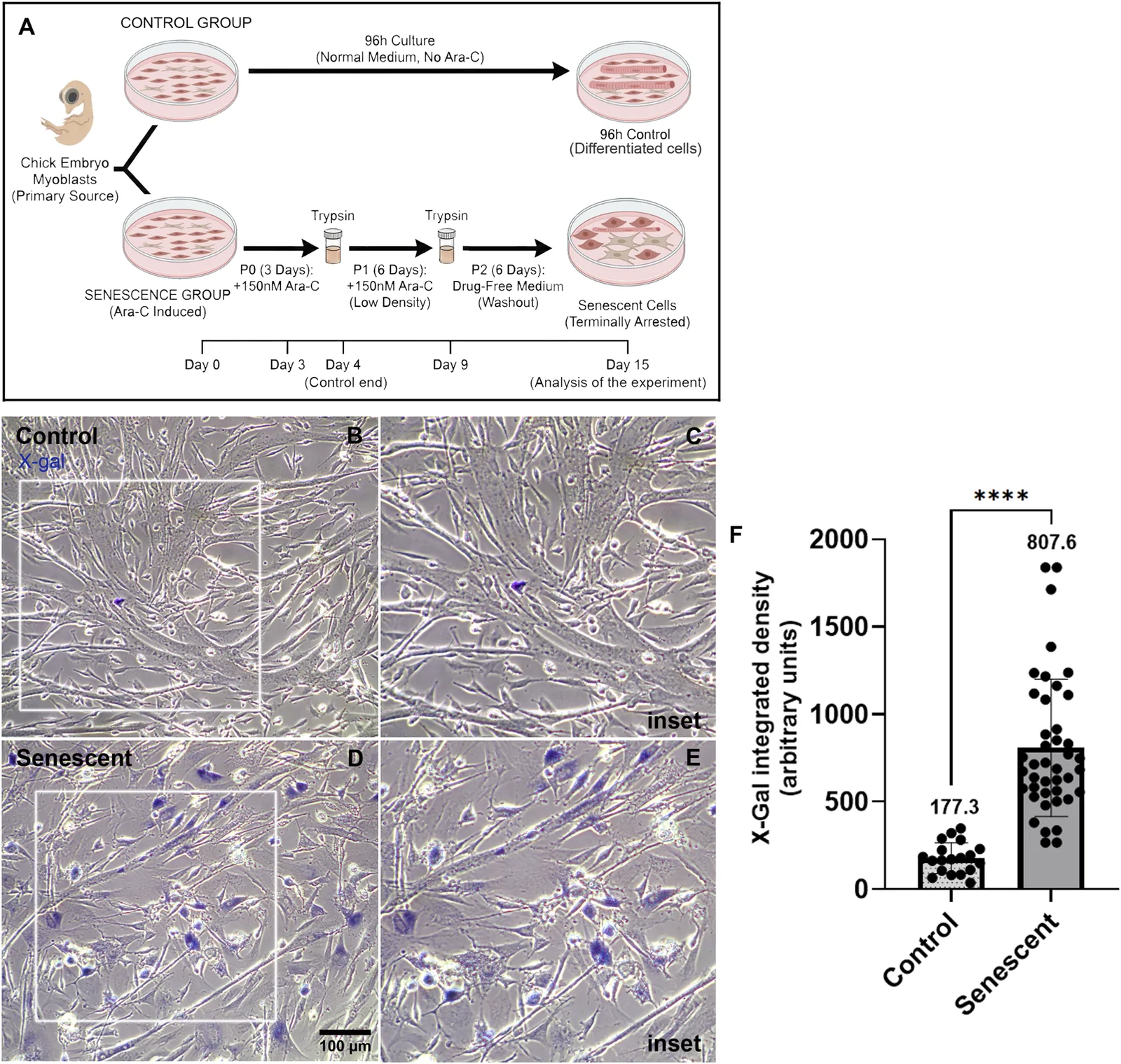
Low doses of Ara-C increases beta-galactosidase (β-gal) expression in chick myogenic cells grown in culture. Primary cultures of chick myogenic cells were grown for 96 h, treated with 150 nM of Ara-C for 72 h and stained for SA-β-gal **(A–F)**. Scale bar in D = 100 µm. A schematic representation of the induction of senescence in skeletal muscle cells is shown in **(A)**. Image in **(C)** is a higher magnification of the inset shown in **(B)**, and image in **(E)** is a higher magnification of the inset shown in **(D)**. A high increase in β-gal expression (in purple) can be observed in myogenic cells after treatment with 150 nM Ara-C **(D–F)**.

**FIGURE 2:**
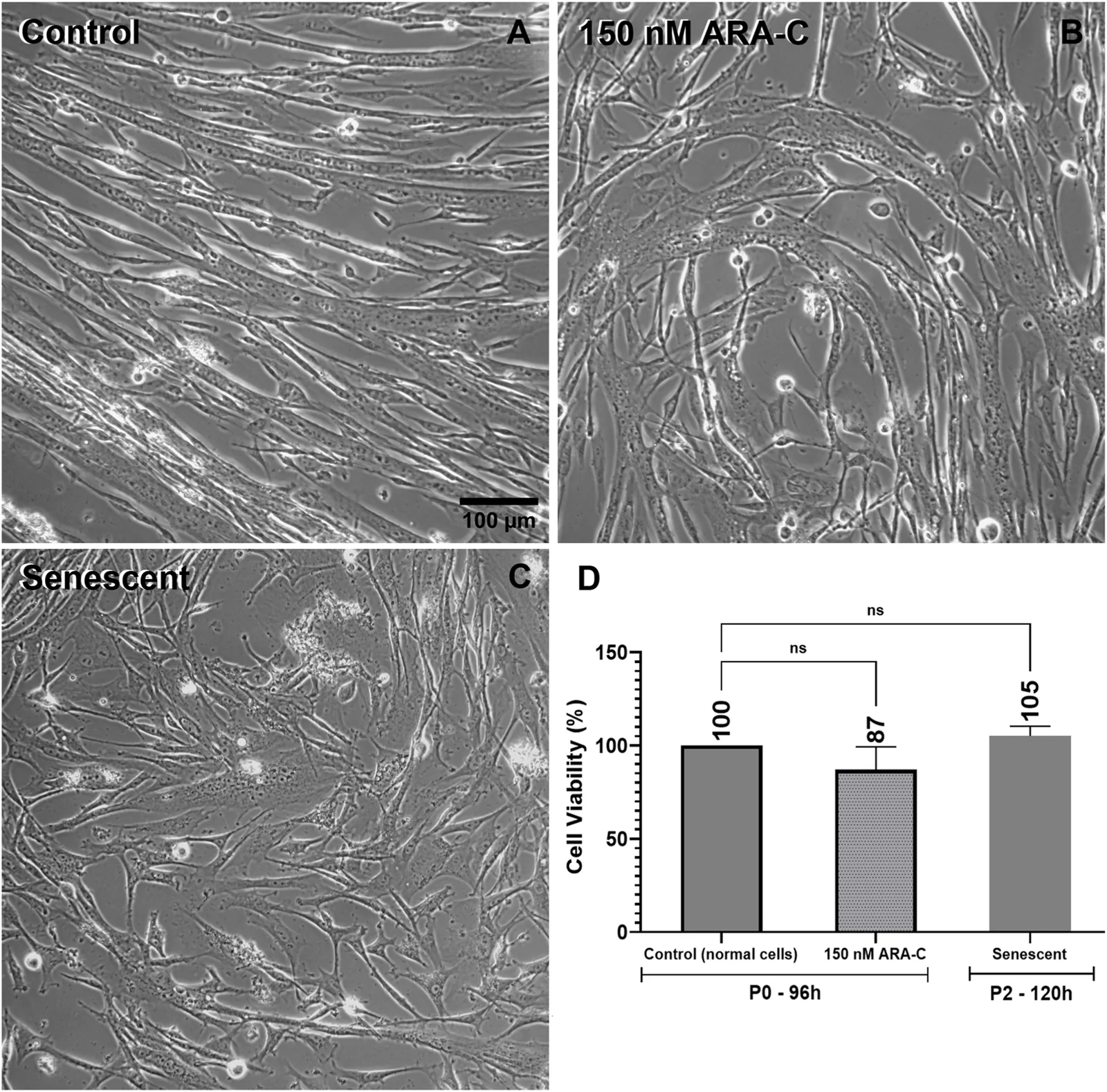
Low doses of Ara-C does not interfere with cell viability of chick myogenic cells grown in culture. Cells were treated with 150 nM Ara-C for 3 days during primary culture (P0). Then, cells were trypsinized and plated again for 6 days (P1), washed and maintained in culture medium without Ara-C for the next 6 days (P2). Cells were observed at phase contrast microscopy **(A–C)**. A = control cell culture, B = P0 cell culture, C = P2 cell culture. Scale bar in A = 100 µm. MTT assay showed no significant changes in cell viability after treatment of cells with low dose of Ara-C in P0 and P2 cultures. n. s = non-significant. MTT assay showed no significant changes in cell viability after treatment of cells with low dose of Ara-C in P0 and P2 cultures **(D)**.

Cellular senescence has been associated with several alterations in the morphology and function of cells (Kumari and Jat, 2021; Ogrodnik et al., 2024). To have a glimpse of the changes that occur in chick myogenic cells undergoing senescence, we investigated several cellular and molecular characteristics, such as the overall cell size and morphology, expression of muscle differentiation markers, expression of p53 and p21, reactive oxygen species (ROS), acid organelles and lysosomes, proliferation markers, and secretion of several cytokines, including IL-6, TNF-α and TGF-β.

Initially, we analyzed the effects of senescence in cell size and cell morphology in the 3 cell phenotypes (myoblasts, myotubes and fibroblasts) found in chick myogenic cell cultures. Cells were triple labelled with antibodies against desmin (muscle-specific marker, Costa et al., 2004) and flotillin-2 (a marker of chick muscle fibroblasts, Possidonio et al., 2014) and with the nuclear dye DAPI (Figure 3). Triple labelling these cells allow us to identify myotubes (positive for desmin, weakly labeled for flotillin-2, and multinucleated), myoblasts (positive for desmin, weakly labeled for flotillin-2, and mononucleated), and fibroblasts (negative for desmin, strongly labeled for flotillin-2, and mononucleated). We observed an increase in the size of myoblasts and fibroblasts and an increase in the number of nuclei in fibroblast cells in senescence cultures (Figures 4, 5), while myotubes seem to be smaller and with less nuclei as compared to control cultures (Figures 1, 3, 4, 5). Our results showing muscle fiber atrophy after Ara-C treatment agree with earlier studies showing that progression of muscle atrophy is particularly accelerated during muscle senescence (Rowan et al., 2011). The increase in the size of muscle fibroblasts after Ara-C treatment is clear in Figure 4, where chick myogenic cells were labeled with the F-actin fluorescent probe Phalloidin and with an antibody against α-tubulin (Figures 4A–L). Interestingly, an increase in actin-containing stress fibers is observed in senescent muscle fibroblasts (Figure 4J), which is in accordance with previous studies (Chen et al., 2000). Quantification of several myogenesis parameters showed that Ara-C treatment induced a decrease in myotube diameter, an increase in myoblast and fibroblast size, an increase in binucleated fibroblasts, and a decrease in myoblast fusion index (Figures 5C–K). The collection of these results indicates a decrease in muscle differentiation during senescence. Confirming these results, we observed a decrease in desmin protein expression in myogenic senescent cells by immunoblotting analysis (Figures 6A,D).

**FIGURE 3:**
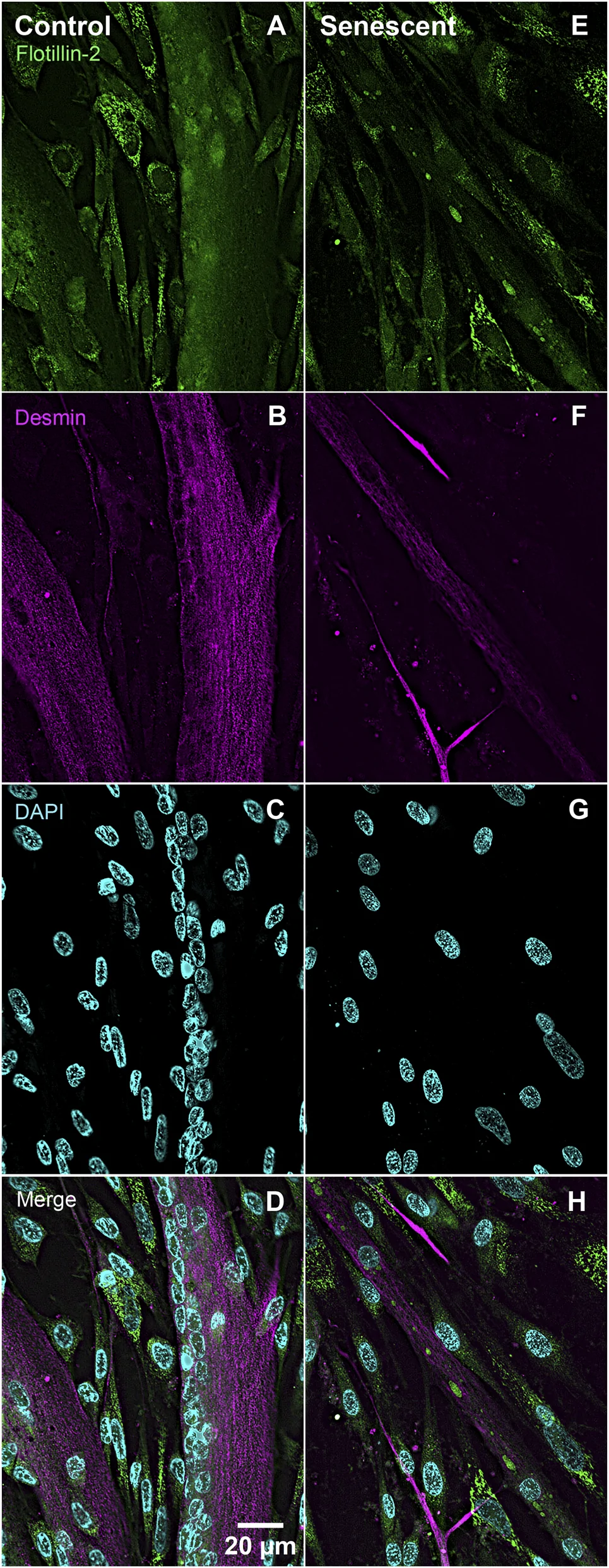
Low doses of Ara-C induce a decrease in the size of myotubes in chick myogenic cells. Primary cultures of chick myogenic cells were grown for 96 h, treated with 150 nM of Ara-C for 72 h and labeled with antibodies against flotillin-2 (green) and desmin (magenta), and with the nuclear dye DAPI **(A–H)**. Merged images are shown in **(D,H)**. Scale bar in D = 20 µm. Note the decrease in the size of myotubes after Ara-C treatment **(F–H)**.

**FIGURE 4:**
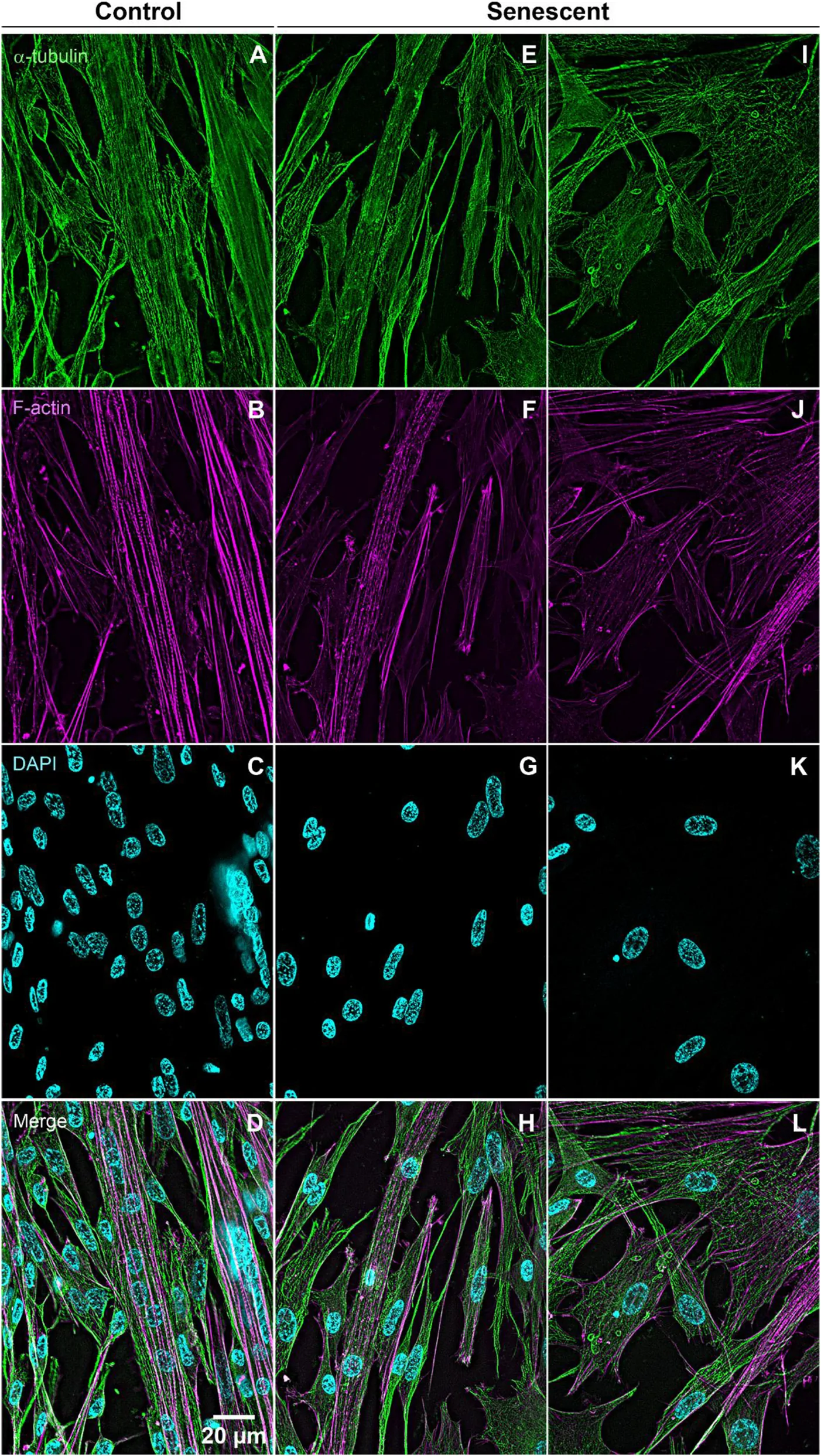
Low doses of Ara-C induce an increase in the size of fibroblasts in chick myogenic cells. Primary cultures of chick myogenic cells were grown for 96 h, treated with 150 nM of Ara-C for 72 h and labeled with an antibody against alpha-tubulin (green), Phalloidin (magenta) and DAPI **(A–L)**. Merged images are shown in **(D,H,L)**. Scale bar in D = 20 µm. Note the increase in the size of fibroblasts after Ara-C treatment **(I–L)**.

**FIGURE 5:**
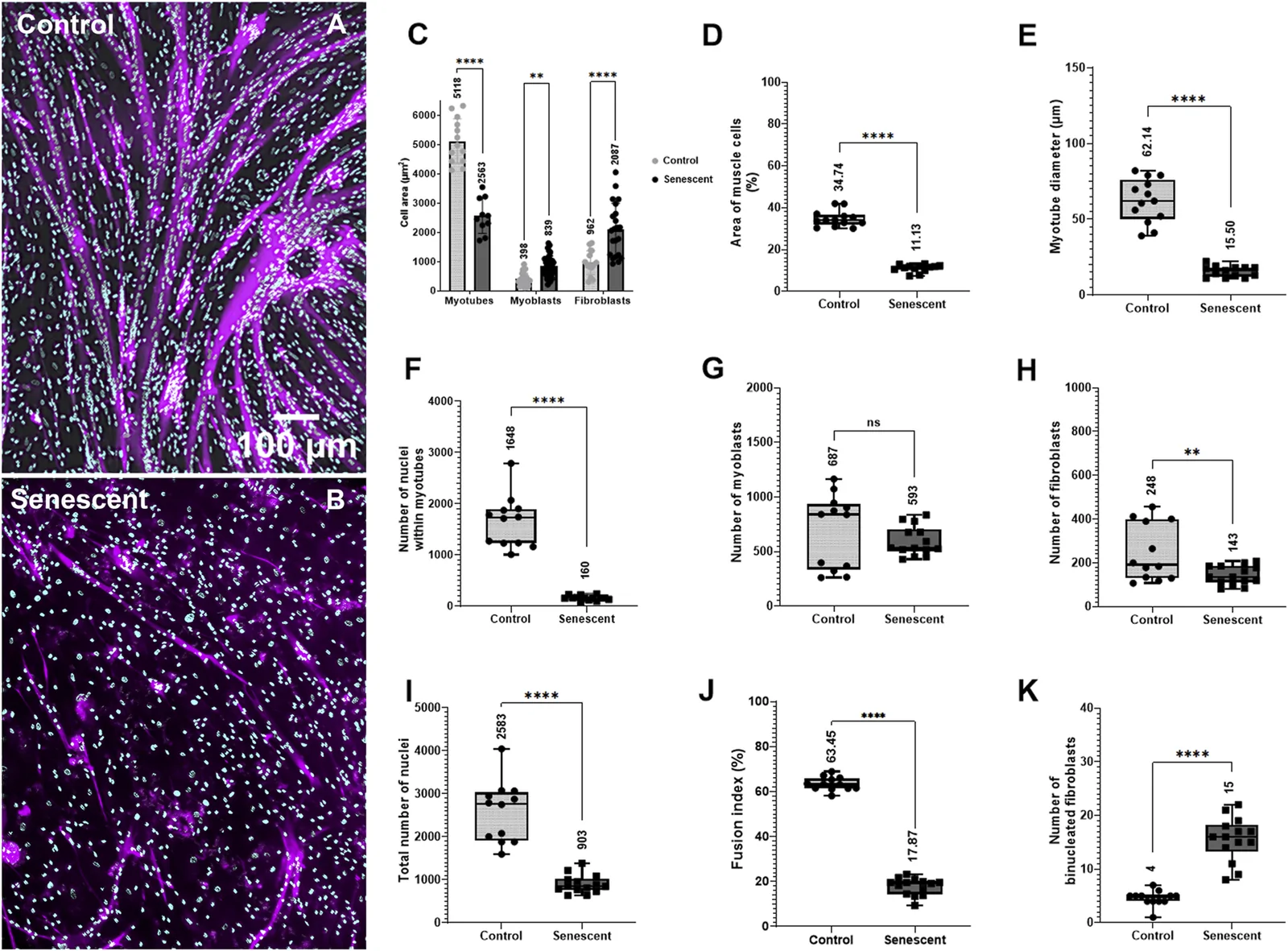
Low doses of Ara-C induce a decrease in myotube size and in myoblast fusion index. Chick myogenic cells were immunolabeled for the muscle-specific marker desmin (magenta, **(A,B)** and the nuclear dye DAPI (cyan, **(A,B)**. Scale bar in A = 100 µm. The quantification of the number of myoblasts, number of fibroblasts, number of nuclei within myotubes, total number of nuclei, myoblast fusion index, cell area, area of muscle cells, myotube diameter and number of binucleated fibroblasts was performed in untreated (control) and AraC-treated cells **(C–K)**. n. s = non-significant.

**FIGURE 6:**
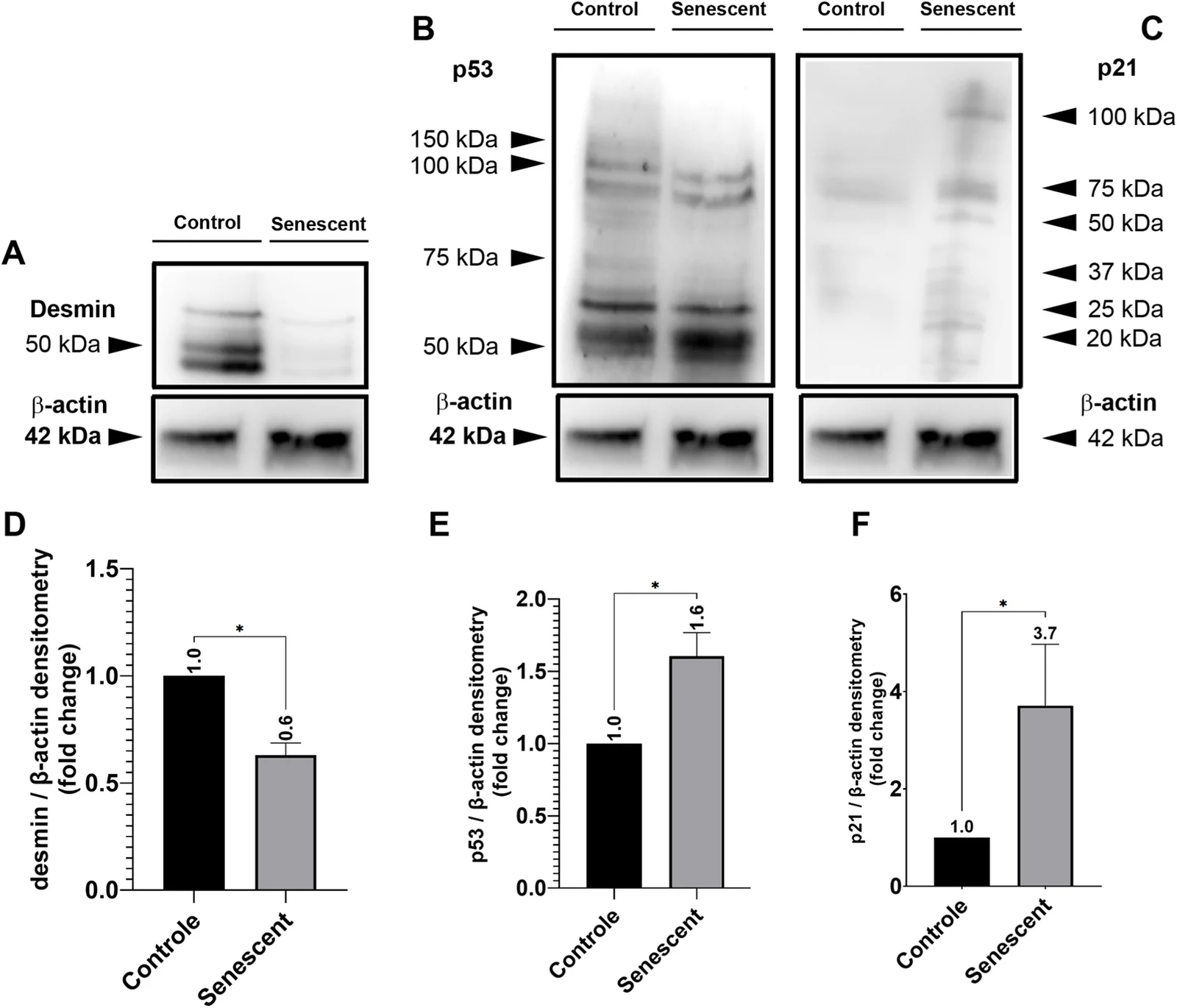
Western blot analysis of the expression of desmin, p53 and p21 in chick myogenic cells treated with Ara-C. Primary cultures of chick myogenic cells were grown for 96 h, treated with 150 nM of Ara-C for 72 h and total protein homogenate were submitted to Western blot for desmin **(A)**, p53 **(B)** and p21 **(C)**. Note the decrease in desmin expression and the increase in p53 and p21 expression after Ara-C treatment **(A–F)**.

Next, we analyzed the expression of p53 and p21 in senescent muscle cells, since both proteins have been associated with cellular senescence due to their ability to trigger cell cycle arrest (Stein et al., 1999; Xue et al., 2007). Immunofluorescence microscopy showed an increase in p53 protein expression in senescent myogenic cells, compared to control cultures (Figure 7). Western blot analysis showed an increase in expression of p21 and p53 (Figures 6B,C,E,F). To evaluate another cell cycle marker, we labeled cells with an antibody against phospho-Histone H3 (pH3), a marker of cell division. Immunofluorescence showed a prominent decrease in pH3 labeling in senescent myogenic cells (Figure 8), indicating a decrease in cell proliferation.

**FIGURE 7:**
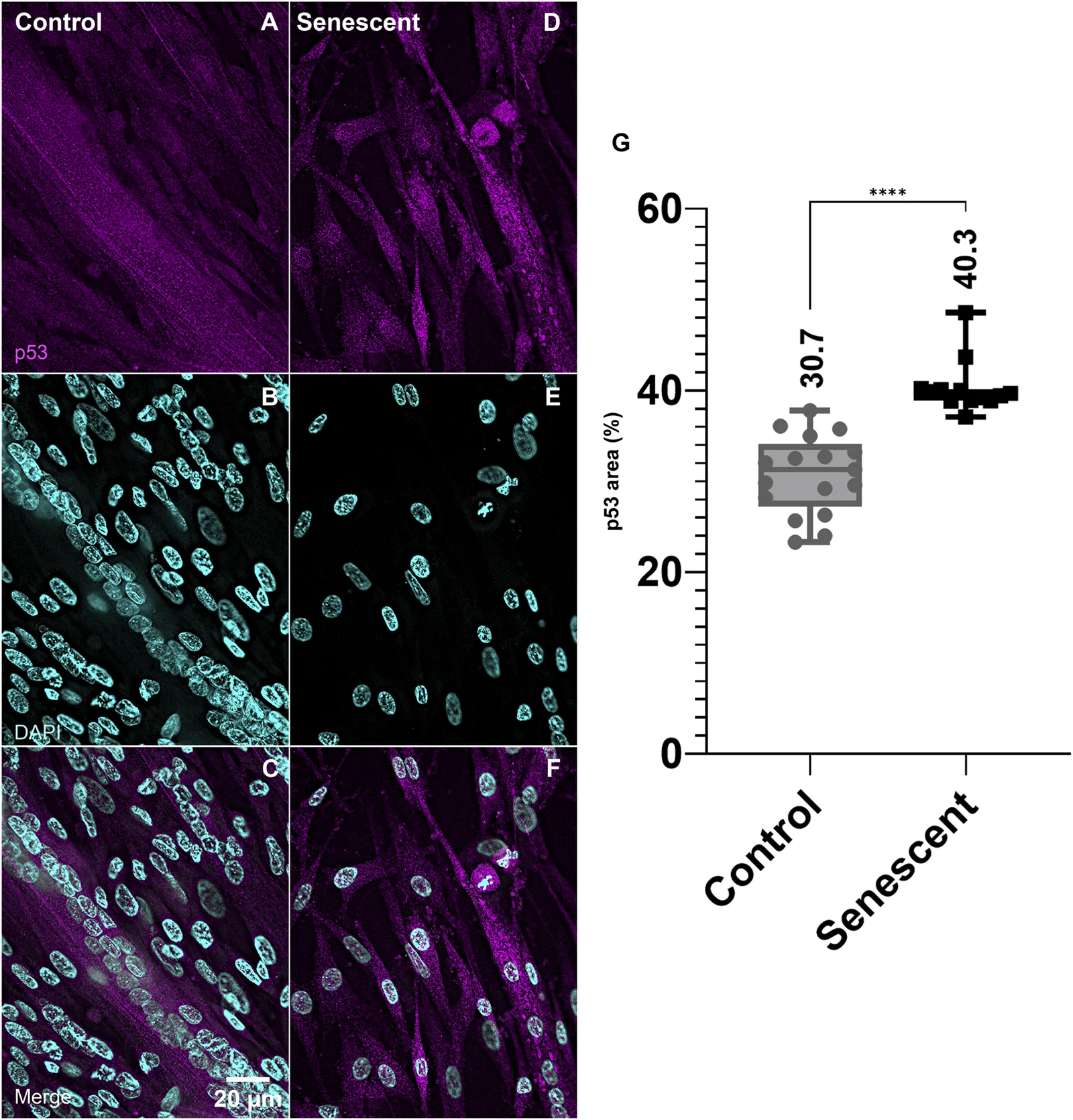
Low doses of Ara-C increase p53 expression in chick myogenic cells grown in culture. Primary cultures of chick myogenic cells were grown for 96 h, treated with 150 nM of Ara-C for 72 h and labeled with an antibody against p53 (magenta) and the nuclear dye DAPI (cyan). Scale bar in C = 20 µm. An increase in p53 labeling can be observed in myogenic cells after treatment with 150 nM Ara-C **(A–G)**.

**FIGURE 8:**
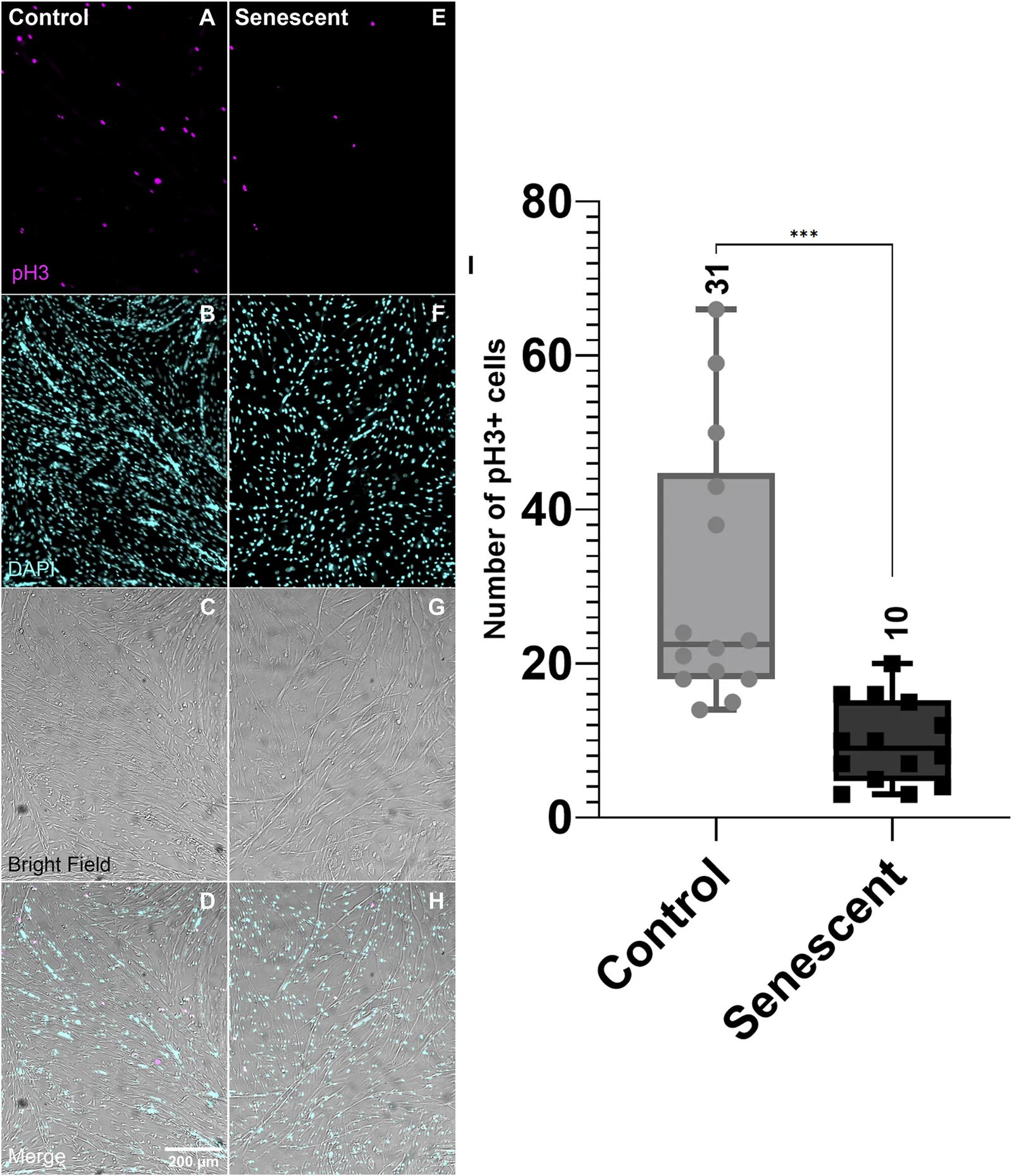
Low doses of Ara-C decrease the number of phospho-histone H3 in chick myogenic cells. Primary cultures of chick myogenic cells were grown for 96 h, treated with 150 nM of Ara-C for 72 h and labeled with an antibody against phospho-histone H3 (pH3, magenta) and DAPI (cyan). Scale bar in D = 200 µm. Note the decrease in the number of pH3-positive cells after Ara-C treatment **(A–I)**.

Another hallmark of senescence is an increase in acidic organelles, including lysosomes (Tan and Finkel, 2023). Using Acridine orange (AO) and Lysotracker we noted a high increase in the labeling of these fluorescence dyes in cells treated with Ara-C (Figures 9, 10), indicating an increase in acidic compartments during muscle senescence. The increase in AO was mainly observed in the red fluorescence wavelength (Figure 9). AO is a fluorescent dye that binds to nucleic acids and acidic compartments in cells, producing different colors (green for DNA, red for RNA/acidic areas) under a fluorescent microscope. Therefore, the red signal of AO indicates an increase in acidic compartments in senescence myogenic cells. Lysotracker labeling showed an increase in muscle senescent cells, compared with control cells. Interestingly, an intense Lysotracker labeling was found in senescent multinucleated myotubes (Figure 10).

**FIGURE 9:**
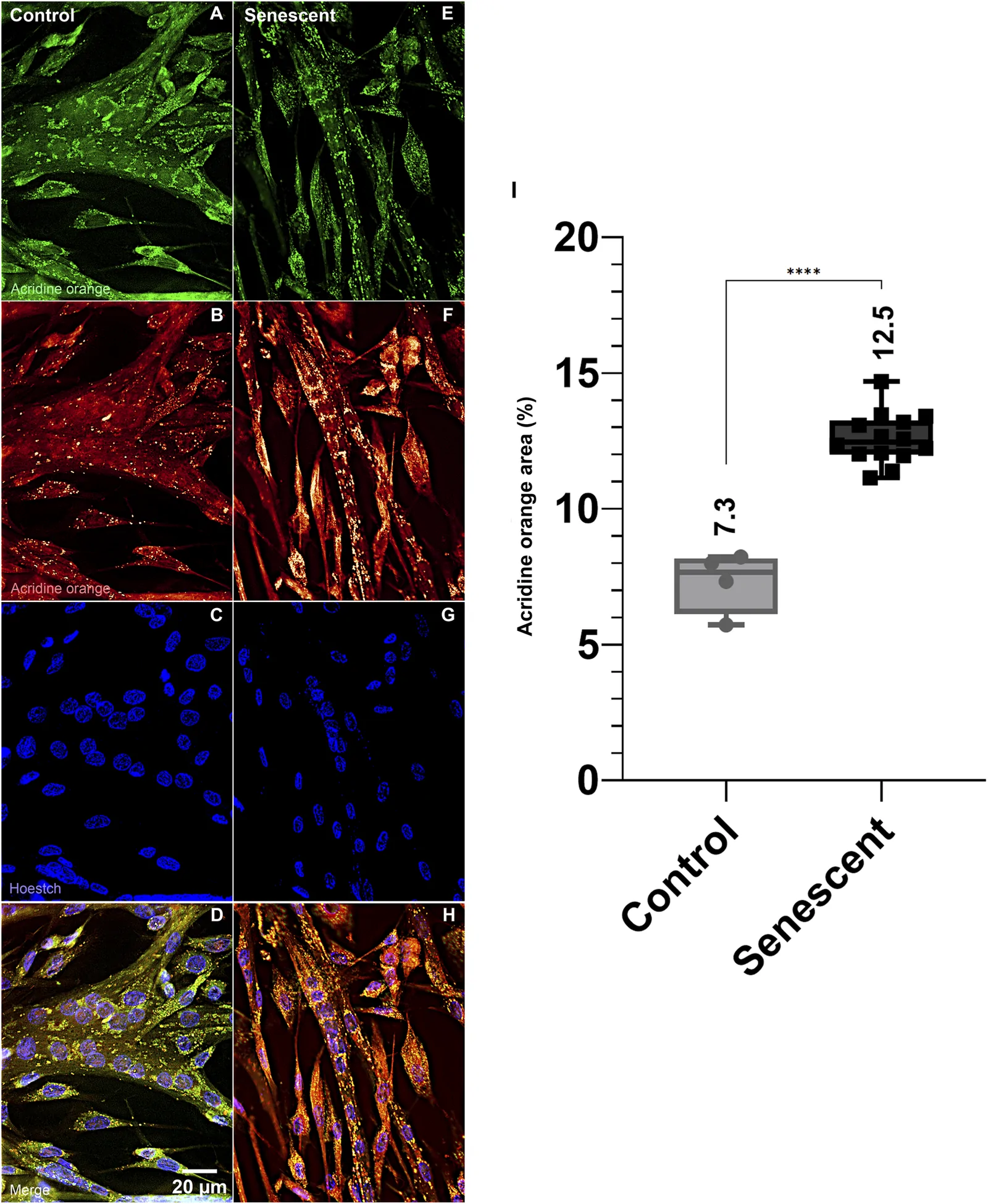
Low doses of Ara-C increase acidic compartments in chick myogenic cells grown in culture. Primary cultures of chick myogenic cells were grown for 96 h, treated with 150 nM of Ara-C for 72 h and labeled with the fluorescent probe Acridine Orange (green and red) and the nuclear dye Hoechst (blue). Scale bar in D = 20 µm. An increase in red fluorescence of Acridine Orange in AraC-treated cells indicates an increase in acidic compartments in myogenic cells **(A–I)**.

**FIGURE 10:**
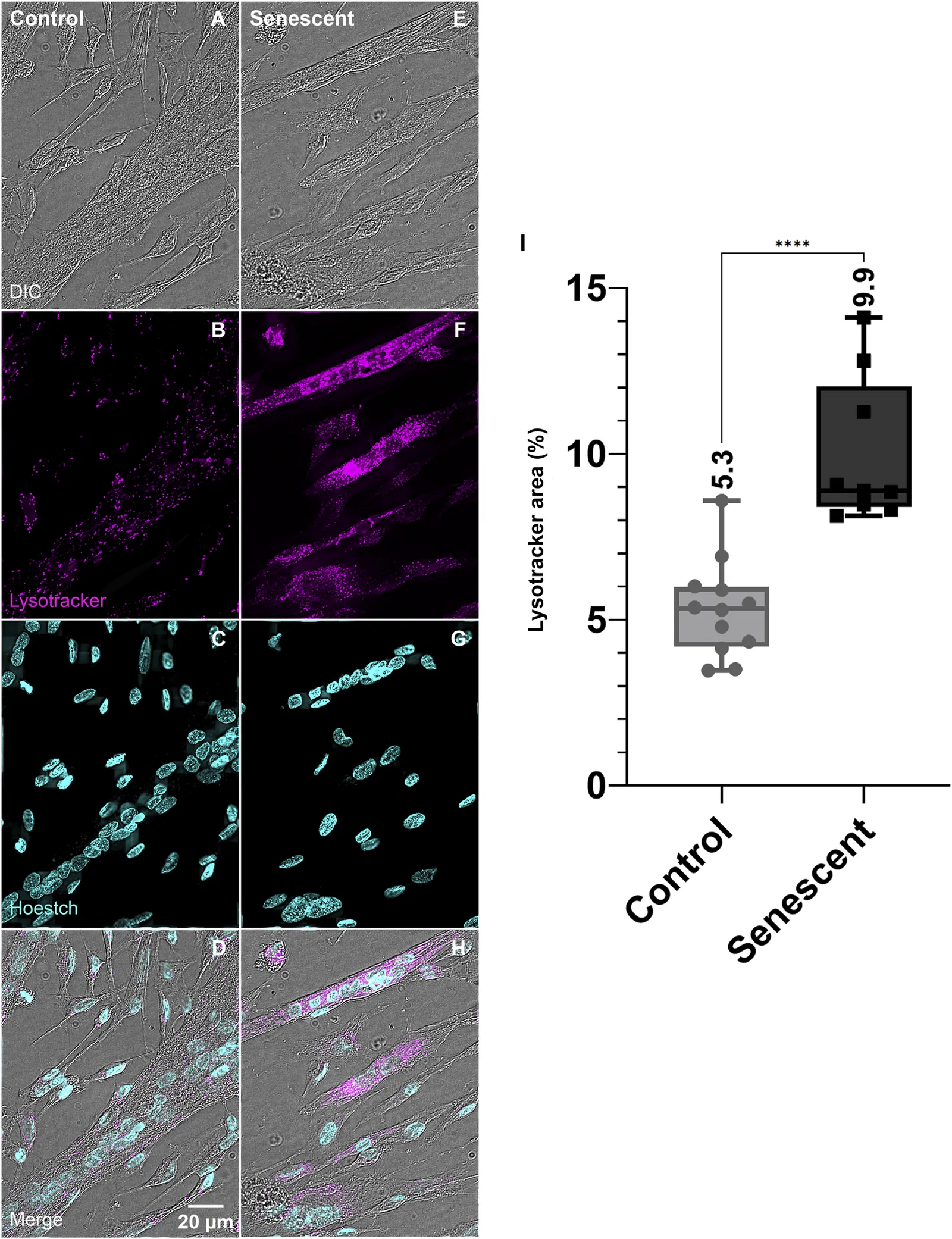
Low doses of Ara-C increase lysosomal activity in chick myogenic cells grown in culture. Primary cultures of chick myogenic cells were grown for 96 h, treated with 150 nM of Ara-C for 72 h and labeled with the fluorescent probe LysoTracker (magenta) and the nuclear dye Hoechst (cyan). Scale bar in D = 20 µm. An increase in LysoTracker labeling can be observed in myogenic cells after treatment with 150 nM Ara-C **(A–I)**.

An increase in reactive oxygen species (ROS) is commonly observed in senescent cells (von Zglinicki, 2024). Therefore, we labeled live chick myogenic cells with the fluorescent probe H2DCFDA to investigate ROS production during muscle senescence. A remarkable increase in ROS was observed in myogenic cells treated with Ara-C (Figure 11). Interestingly, Palomero and colleagues (2013) reported that isolated muscle fibers from old mice generate more ROS than young adult mice, and that increased ROS production is a potent promoter of cellular senescence.

**FIGURE 11:**
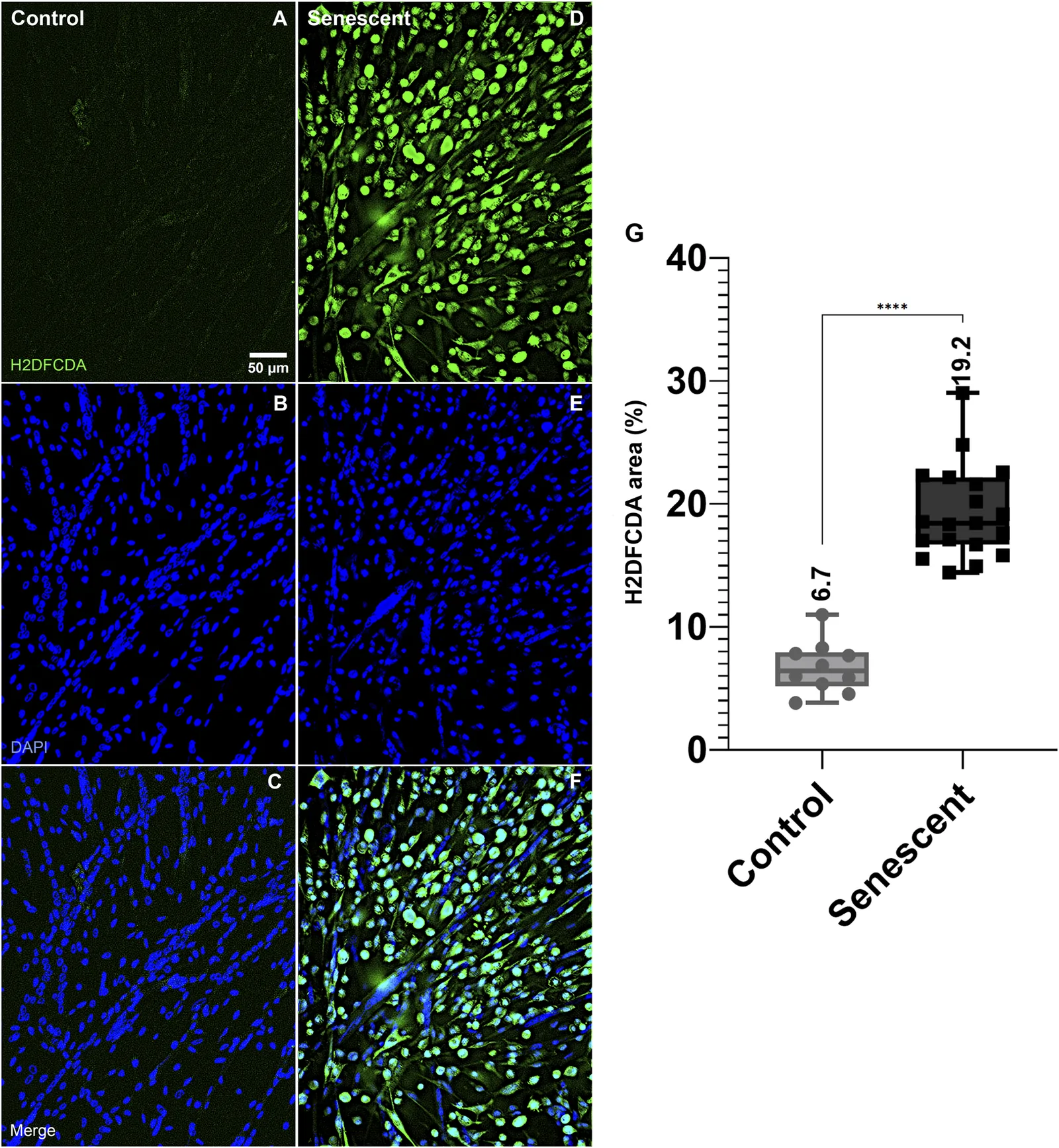
Low doses of Ara-C increase Reactive Oxygen Species (ROS) in chick myogenic cells grown in culture. Primary cultures of chick myogenic cells were grown for 96 h, treated with 150 nM of Ara-C for 72 h and labeled with ROS fluorescent probe H2DCFDA (green) and the nuclear dye DAPI (blue). Scale bar in A = 50 µm. A high increase in ROS can be observed in myogenic cells after treatment with 150 nM Ara-C **(A–G)**.

To investigate the penetrance of the senescent phenotype in myogenic cultures we stained cells with Sudan Black B, a known marker of lipids that is usually increased during cellular senescence (Evangelou and Gorgoulis, 2017). Sudan Black B staining was assessed by bright field (BF) and fluorescence microscopies, and an intense dark blue staining was observed under bright field, and an intense red fluorescence was noted in muscle senescence cells (Figure 12).

**FIGURE 12:**
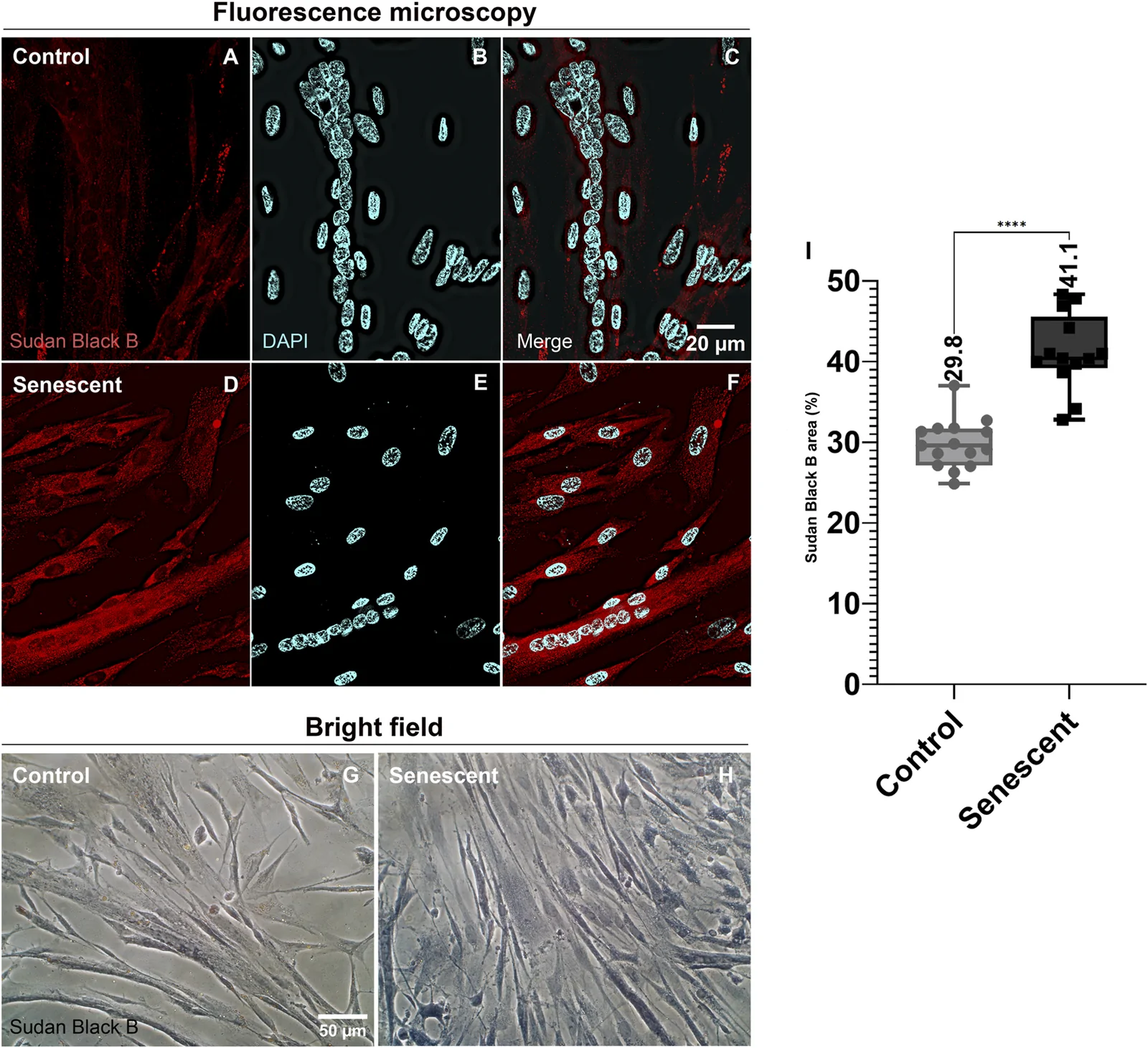
Low doses of Ara-C increases Sudan Black staining in chick myogenic cells grown in culture. Primary cultures of chick myogenic cells were grown for 96 h, treated with 150 nM of Ara-C for 72 h and stained with Sudan Black (red) and the nuclear dye DAPI (cyan). After Sudan Black staining, cells were observed under fluorescence microscopy (A–F) and under bright field microscopy (G–H). Scale bars = 20 µm in (C) and 50 µm in (G). A high increase in Sudan black stain can be observed in myogenic cells after treatment with 150 nM Ara-C **(A–I)**.

Finally, we analyzed the senescence-associated secretory phenotype (SASP) of chick myogenic cells treated with Ara-C (Basilicata et al., 2025). ELISA assay showed an increase in the secretion of IL-6, TNFα and TGFβ in the conditioned media of Ara-C-treated cells (Figure 13). TGF-β1 is well-known as a major cytokine involved in the regulation of cellular senescence (Tominaga and Suzuki, 2019) and it can cause oxidative stress-induced activation of the p53 pathway and senescence (Samarakoon et al., 2019). Furthermore, our results agree with previous studies reporting that IL-6 induces skeletal muscle atrophy (Haddad et al., 2005). Interestingly, it has been shown that IL-6, a key SASP factor, has the potential to amplify cellular senescence in SASP-producing cells in an autocrine action (Herbstein et al., 2024).

**FIGURE 13:**
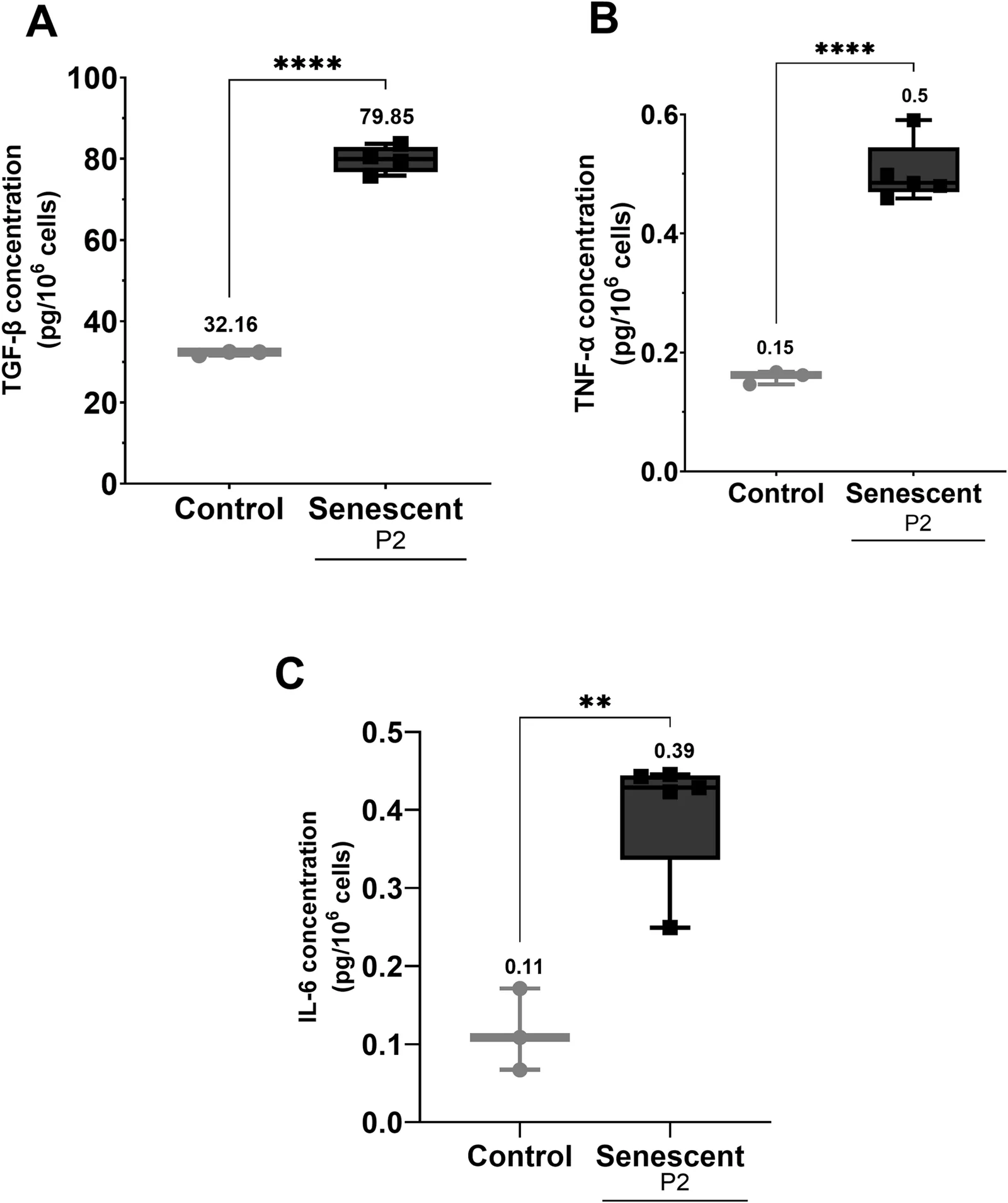
Low doses of Ara-C induce TGFβ, TNFα and IL-6 secretion in chick myogenic cells grown in culture. Primary cultures of chick myogenic cells were grown for 96 h, treated with 150 nM of Ara-C for 72 h and the conditioned media was collected. The concentration of TGFβ, TNFα and IL-6 were analyzed by ELISA **(A–C)**. Note the increase in the concentration of all the cytokines in conditioned media after Ara-C treatment.

The collection of our results shows that Ara-C at low doses induce muscle cell senescence in embryonic chick myogenic cell cultures. The senescence phenotype was confirmed by the following results: (i) increased β-galactosidase, (ii) increased lipids (Sudan Black B), (iii) increased ROS, (iv) increased p53 and p21 expression, (v) increased secretion of IL-6, TNFα and TGFβ, (vi) increased lysosomal activity, (vii) decreased cell proliferation, (viii) decreased muscle fiber size, (ix) decreased desmin protein expression, (x) increased fiber stress in fibroblasts, and (xi) fibroblasts with increased size and nuclei numbers. A proposed working model of the changes that occur during skeletal muscle senescence as well as the muscle senescence-associated traits observed in our study is illustrated in Figure 14. The relationship between these traits are complex and intertangled. Nonetheless, we suggest the following: Ara-C induces cell cycle arrest, which in turn activates p53, who activates the senescent program, including alterations in lysosomal and mitochondrial physiology, leading to increased ROS production, and to an increase in beta-galactosidase activity and SASP. All these responses inhibit muscle differentiation.

**FIGURE 14:**
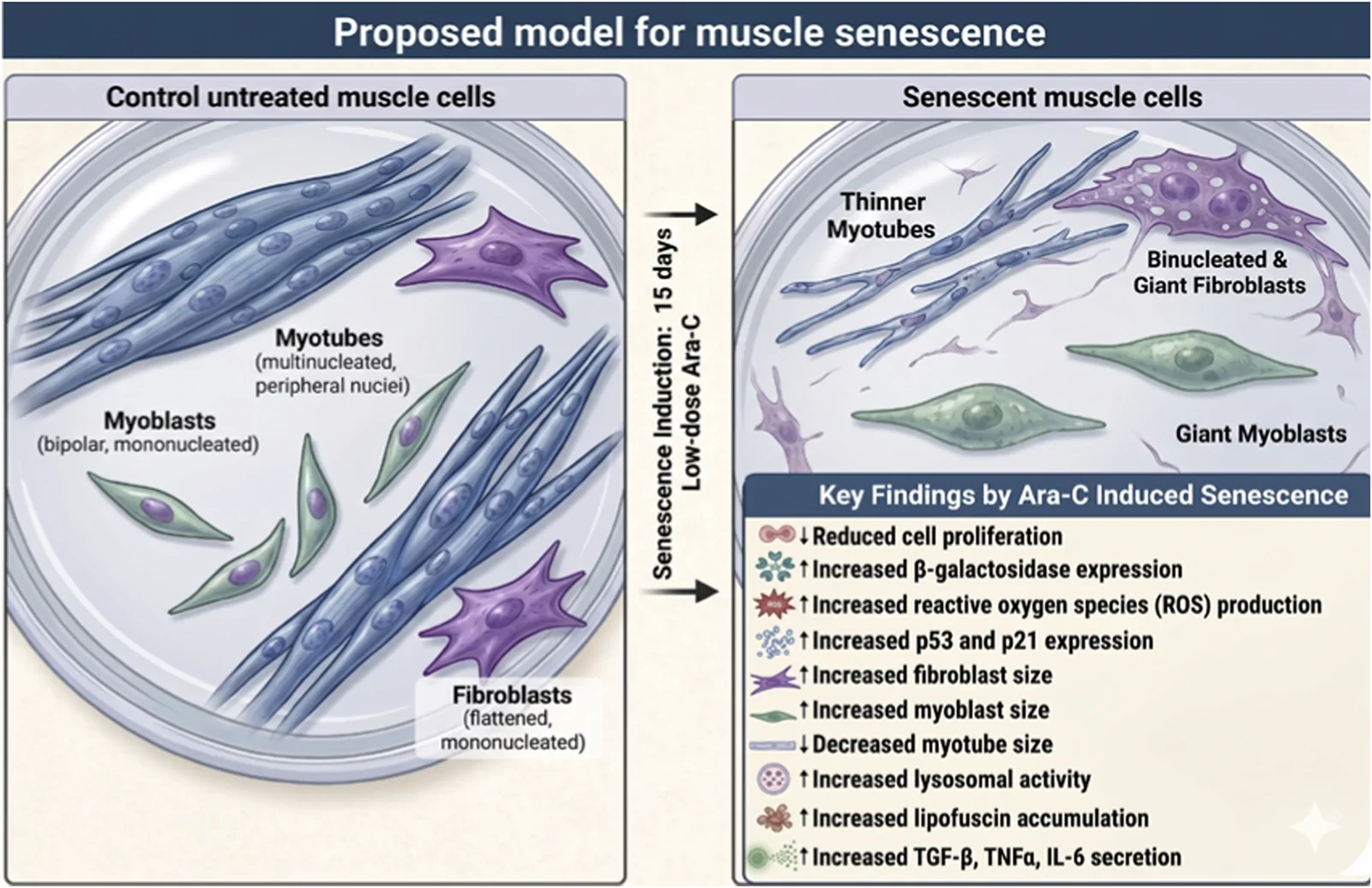
Proposed working model for skeletal muscle cell senescence. Schematic representation of a proposed working model for muscle senescence, including the changes in cellular morphology and protein expression that occur during the process. Myotubes are represented in blue, myoblasts in green and fibroblasts in magenta.

Remarkably, our data points to a difficulty to distinguish between cellular senescence and cellular stress in the AraC-induced phenotype in muscle cells. As noted by Ben-Porath and Weinberg (2004), cellular senescence should be viewed in terms of its role as a general cellular stress response program, rather than strictly as a barrier to unlimited cycles of cell growth and division. In agreement with this view, we found increased ROS production, SASP, and higher lysosomal activity in AraC-induced phenotype, which are all cellular stress markers. Nonetheless, AraC is a well-known inducer of cell cycle arrest, which is a hallmark of cellular senescence. Thus, our data highlights how senescence and cellular stress phenotypes are interconnected.

Finally, to gain further insight into the mechanism implicated in the establishment of muscle cell senescence phenotype, we decided to test whether the Fibroblast Growth Factor (FGF) signaling pathway was involved in this process. FGF was selected because of its previously described role in the regulation of cellular senescence and aging in skeletal muscle (Mathes et al., 2021; Pawlikowski et al., 2017; Sato et al., 2016). Furthermore, our results showed that AraC-induced senescence led to the formation of giant fibroblasts in chick myogenic cell cultures (Figure 4), pointing to a possible role of a fibroblast-related signaling pathway in the process. Control (untreated) and senescent-induced muscle cells were treated with SU-5402, an inhibitor of FGF receptor (FGFr). Our results show that SU-5402 was able to recover the AraC-induced senescent phenotype in myogenic cells, as observed by the increase in the formation of muscle fibers (Figure 15). These results suggest that FGF is involved in the induction of senescent phenotype in chick muscle cells and that its inhibition can, at least partially, prevent muscle cell senescence. Further studies are necessary to dissect the molecular mechanisms involved in FGFR signaling during muscle senescence.

**FIGURE 15:**
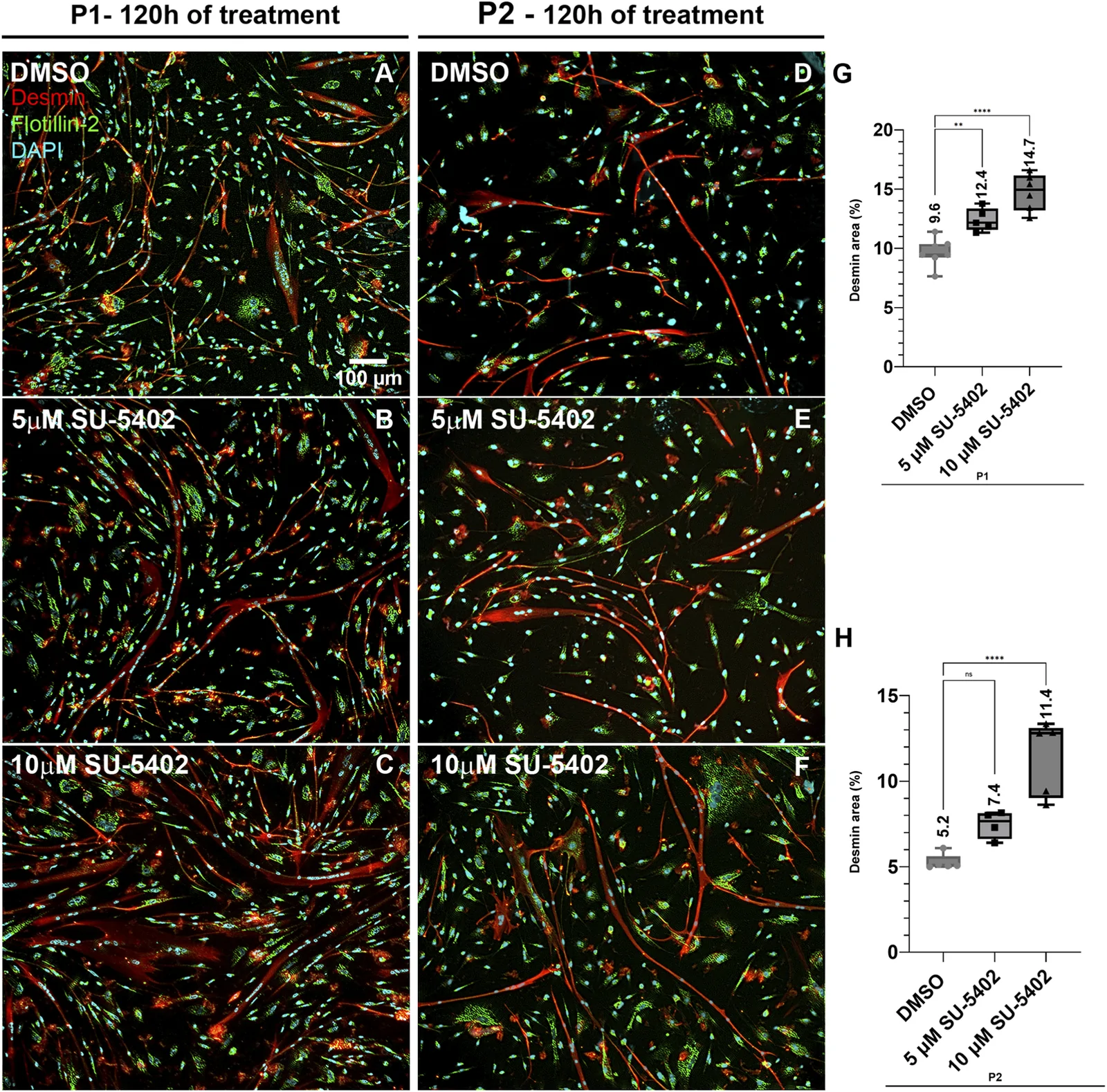
Inhibition of FGFr rescues AraC-induced senescence phenotype in chick myogenic cells. Primary cultures of chick myogenic cells were grown for 96 h, treated with 150 nM of Ara-C for 72 h and then treated with 5 or 10 µM of SU-5402, an inhibitor of Fibroblast Growth Factor receptor (FGFr). Cells were labeled with antibodies against flotillin-2 (green) desmin (red), and with the nuclear dye DAPI (cyan). Merged images are shown in **(A-F)** Scale bar in A = 100 µm. Quantification of the desmin area shows an increase in muscle cell fibers (myotubes) after inhibition of FGFr **(G,H)**. For detailed information about P1 and P2 treatments, see the “Senescence induction” methodology section.

One of the advantages of this *in vitro* senescence model is its similarity with the *in vivo* muscle environment since muscle tissue is composed of muscle cells and connective tissue (mainly fibroblasts) and the primary culture of chick myogenic cells comprises both muscle and fibroblast cells. Another advantage of this model is its simplicity since it uses only one drug (Ara-C) to achieve muscle senescence in 15 days.

One limitation of our study is the lack of a comparison between Ara-C and other known senescence-inducing agent (e.g., ionizing radiation, doxorubicin, H_2_O_2_) in the chick cell system, which we intend to accomplish in future experiments.

This muscle senescence model could be a valuable tool in future studies aiming to investigate the molecules and signaling pathways involved in the initiation and maintenance of the senescence phenotype in several muscle conditions, such as aging, sarcopenia, muscle disuse, and muscle dystrophies.

## Data Availability

The raw data supporting the conclusions of this article will be made available by the authors, without undue reservation.
