# Supplementary material for: Unraveling skeletal muscle senescence in an in vitro cell culture model
Source: Front Aging. 2026 Sep 18;7:1866906. doi: 10.3389/fragi.2026.1866906 (PMC13631421; doi:10.3389/fragi.2026.1866906)
Supplement: Supplementary file 1 [file DataSheet1.pdf]

tools.carbocation.com/FDR

Tools | False Discovery Calculator | Extreme P Value Calculator

False Discovery Rate

Rank	ID	Original P value	Critical Value	Benjamini-Hochberg Adjusted P value	Significant using an FDR of 0.05?
1	1	0.0001	0.007142857142857143	0.00011666666666666668	Yes
2	2	0.0001	0.014285714285714285	0.00011666666666666668	Yes
3	3	0.0001	0.02142857142857143	0.00011666666666666668	Yes
4	5	0.0001	0.02857142857142857	0.00011666666666666668	Yes
5	6	0.0001	0.03571428571428572	0.00011666666666666668	Yes
6	7	0.0001	0.04285714285714286	0.00011666666666666668	Yes
7	4	0.1	0.05	0.1	No

Results of the FDR test with p values from the quantifications shown in Figure 5 (see below).

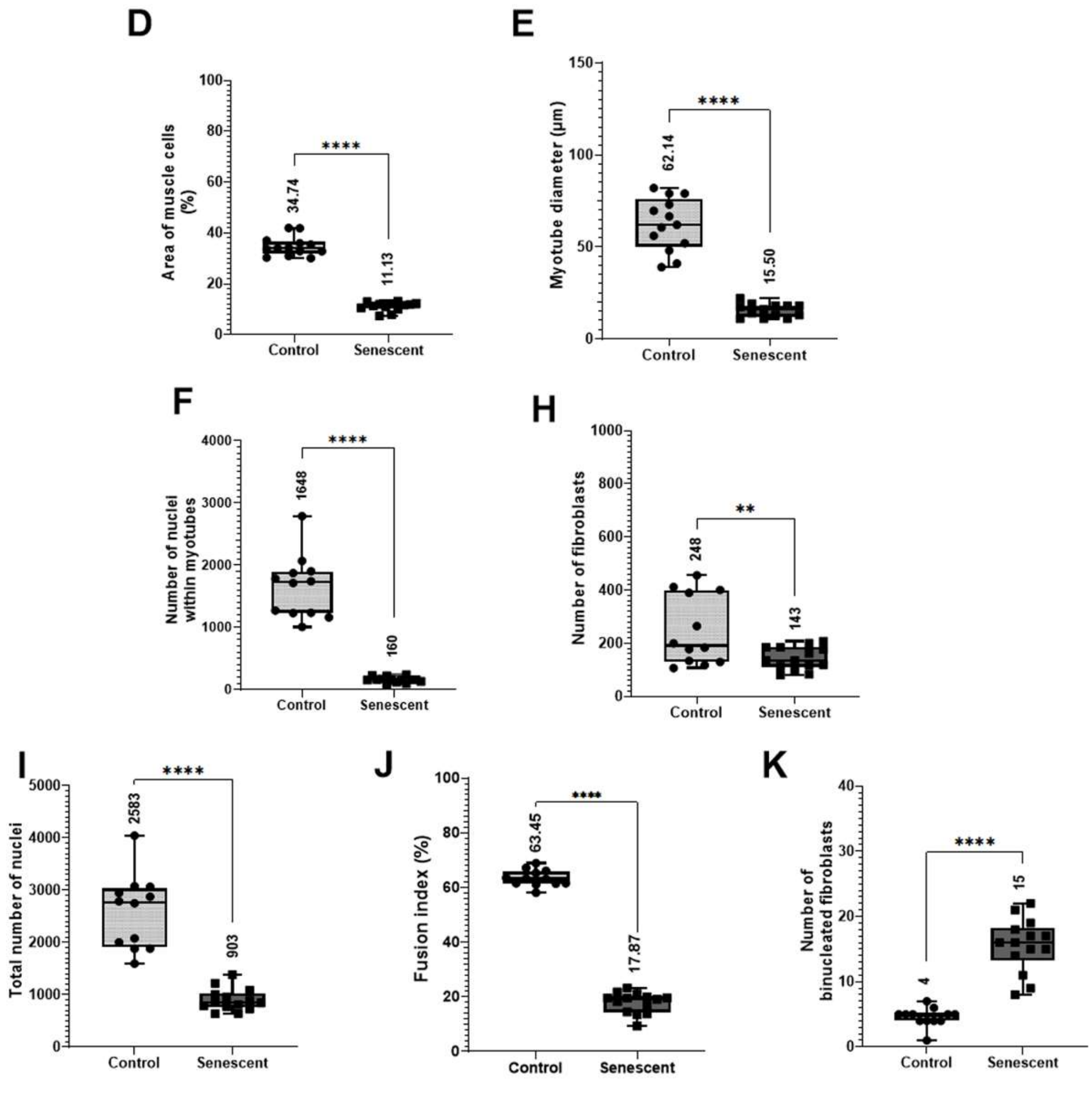

Figure 5 with p values from the quantifications: ** P ≤ 0.01, **** P ≤ 0.0001.
